# Resequencing and SNP discovery of Amur ide (*Leucis*c*us waleckii)* provides insights into local adaptations to extreme environments

**DOI:** 10.1038/s41598-021-84652-5

**Published:** 2021-03-03

**Authors:** Shuangyi Wang, Youyi Kuang, Liqun Liang, Bo Sun, Xuefei Zhao, Limin Zhang, Yumei Chang

**Affiliations:** 1 National and Local United Engineering Laboratory of Freshwater Fish Breeding, Heilongjiang River Fisheries Research Institute, Chinese Academy of Fishery Sciences, Harbin, 150070 China; 2 College of Fisheries and Life Science, Shanghai Ocean University, Shanghai, 200000 China; 3 College of Wildlife and Protected Area, Northeast Forestry University, Harbin, 150040 China

**Keywords:** Protein structure predictions, Population genetics

## Abstract

Amur ide (*Leuciscus waleckii*), a Cyprinid species, is broadly distributed in Northeast Asia. Different from its freshwater counterparts, the population in Lake Dali Nor has a strong alkalinity tolerance and can adapt to extremely alkali–saline water with bicarbonate over 50 mmol/L. To uncover the genetic basis of its alkaline adaptation, three populations, including one alkali form from Lake Dali Nor (DL), one freshwater form from its adjacent sister Lake Ganggeng Nor (GG), and one freshwater form from its historical origin, namely, the Songhua River (SH), were analyzed using genome resequencing technology. A total of 679.82 Gb clean data and 38,091,163 high-quality single-nucleotide polymorphism (SNP) loci were detected in the three populations. Nucleotide diversity and population structure analysis revealed that the DL and GG populations have lower nucleotide diversities and different genetic structures than those of the SH population. Selective sweeping showed 21 genes involved in osmoregulatory regulation (*DLG1*, *VIPR1*, *AKT1*, and *GNAI1*), inflammation and immune responses (*DLG1*, *BRINP1*, *CTSL, TRAF6, AKT1, STAT3, GNAI1, SEC22b,* and *PSME4b*), and cardiorespiratory development (*TRAF6*, *PSME4b*, *STAT3*, *AKT1*, and *COL9A1*) to be associated with alkaline adaption of the DL population. Interestingly, selective pressure (CodeML, MEME, and FEL) methods identified two functional codon sites of *VIPR1* to be under positive selection in the DL population. The subsequent 3D protein modeling confirmed that these selected sites will incur changes in protein structure and function in the DL population. In brief, this study provides molecular evidence of population divergence and alkaline adaptation, which will be very useful for revealing the genetic basis of alkaline adaptation in Amur ide.

## Introduction

Amur ide (*Leuciscus waleckii*) belongs to Cyprinidae and is widely distributed throughout Northeast Asia. This species not only inhabits freshwater but also survives in alkali–saline water^1^. For example, Amur ide can inhabit Lake Dali Nor (116°25′–116°45′E, 43°13′–43°23′N), Inner Mongolia, China, which is a typical alkali–saline lake with HCO_3_^−^/CO_3_^2−^ concentrations greater than 50 mmol/L (pH 9.6) and salinities below 6‰^2,3^. In addition, the alkali Amur ide participates in spawning migration; it spawns in a small freshwater river (Shali River) in late April to early May every year and then returns to Lake Dali Nor for growth^4,5^. Geological and genetic studies have shown that the alkali form originated from the ancient freshwater forms of the Amur River during the early Holocene period^6^. Subsequently, due to the constant and harsh drought in the late Holocene period, Lake Dali Nor shrunk, and the water became seriously alkaline; Amur ide gradually adapted to the alkalinized environment and became the dominant fish species in Lake Dali Nor over the past several thousand years^3,6^.

The adaptation of Amur ide to the extreme alkali–saline environment occurs rapidly, which has represented a local adaptation pattern on a short evolutionary time scale of thousands of years. Previous studies have found that alkali form had stronger alkalinity tolerance and lower genetic diversity than its freshwater forms^2,3^. In recent years, using high-throughput sequencing technologies, transcriptomic expression profiles were compared in Amur ide ecotypes, revealing that many candidate genes associated with alkaline environments were differentially expressed^1,2,4,8^. Moreover, by comparing Amur ide in Lake Dali Nor and its adjacent sister freshwater form in Lake Ganggeng Nor, strong positive selection under alkaline environmental stress was observed for some genes^1^. Subsequently, by combining genome scans with landscape genomic methods, Xu, et al.^9^ found several genomic regions associated with alkaline adaptation under selective sweeps when comparing Amur ide in Lake Dali Nor and its ancestral freshwater form in the Amur River, which are candidate genes involved in processes such as ion homeostasis, reactive oxygen species elimination, and urea excretion. These findings suggest that Amur ide individuals dwelling in Lake Dali Nor have evolved unique genetic strategies that differ from their freshwater counterparts to cope with extremely alkaline environments.

**Figure 1:**
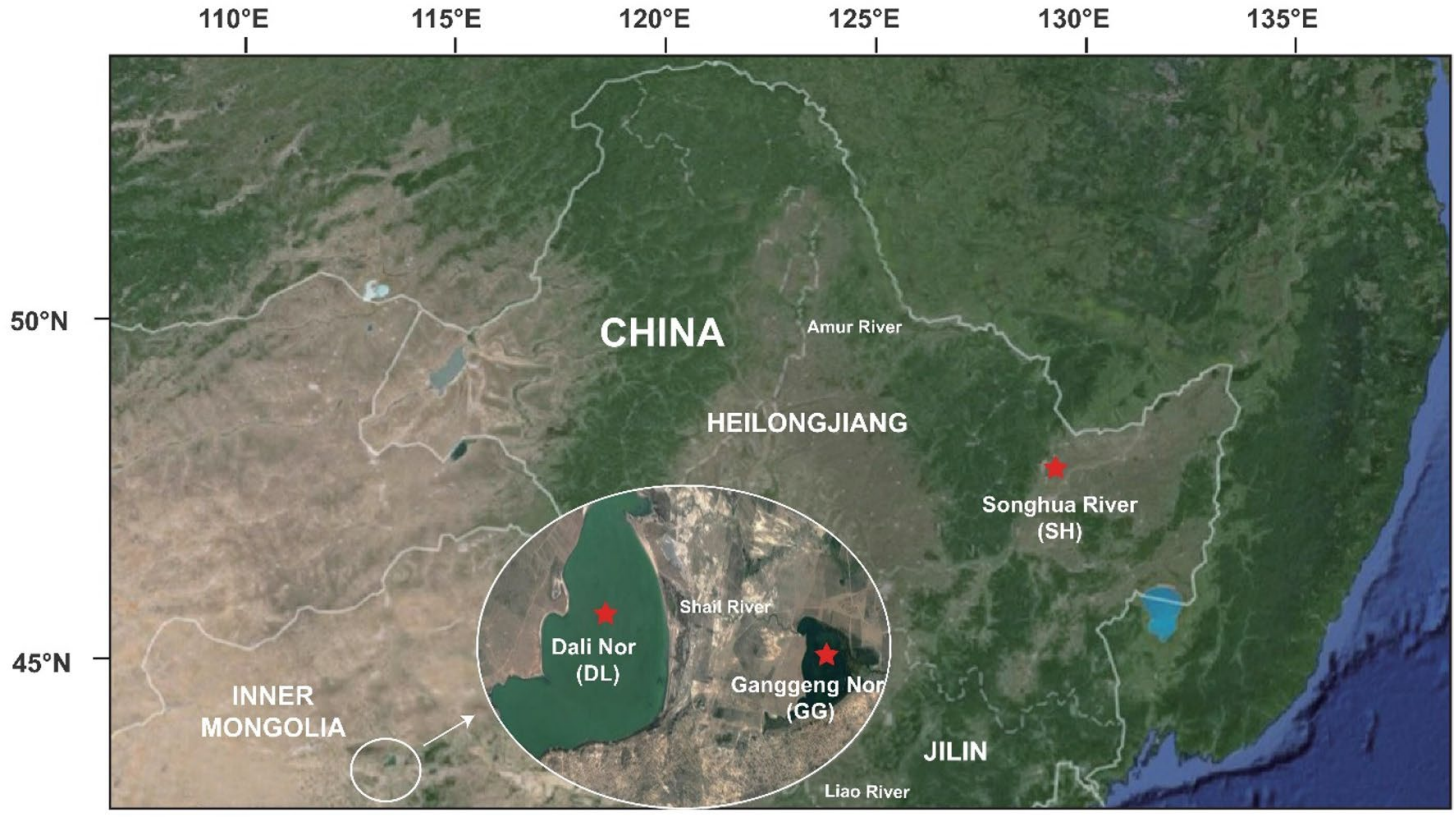
Geographical distribution of the sampled populations (solid red five-pointed stars represent the sampling locations). The map in the background has been generated by R package ‘ggmap’ (https://cran.r-project.org/web/packages/ggmap/)^10^.

Although several of the studies mentioned above reported observed phenotypic and genetic differentiation in Amur ide ecotypes, convincing evidence for the alkaline adaption of Amur ide is still lacking. Chang, et al.^2^ first analyzed six populations from the Amur River and Dali basin using microsatellite DNA markers and suggested that geographic isolation is likely the major force causing population divergence instead of a contrasting environment, demonstrating that it is necessary to combine historical origin and environmental factors to reveal the genetic basis of alkaline adaptation in Amur ide using abundant markers or genomic scans. Despite completely different environments, there are slight genetic differences due to gene flow between populations from Lake Dali Nor and its adjacent freshwater Lake Ganggeng Nor^2,3^. Therefore, to reduce or eliminate the discrepancy of spatial background and to focus on the differences of contrasting environments such as alkaline water and freshwater, we collected three populations, the alkali form from Lake Dali Nor (hereafter abbreviated as DL), the freshwater form from its adjacent sister Lake Ganggeng Nor (hereafter abbreviated as GG) and the freshwater form from its historical origin, the Songhua River (hereafter abbreviated as SH), which is one of the major branches of the Amur River (Fig. 1). Using high-throughput genome resequencing technology, the nucleotide diversity and population structure among the three populations were analyzed based on high-quality SNP calling data. Then, selective sweep analysis was performed to explore candidate genes for alkaline adaptation by comparing populations from contrasting environments. Finally, selective pressure (CodeML, MEME, and FEL) and protein structure analyses were applied to find evidence for adaptive selection in candidate genes from the DL population. This study aims to identify target genes or pathways related to alkaline adaptation and provides new insights into the genetic basis of alkaline adaptation of Amur ide.

## Results

### Resequencing genome profiles, SNP identification and nucleotide diversity

**Figure 2:**
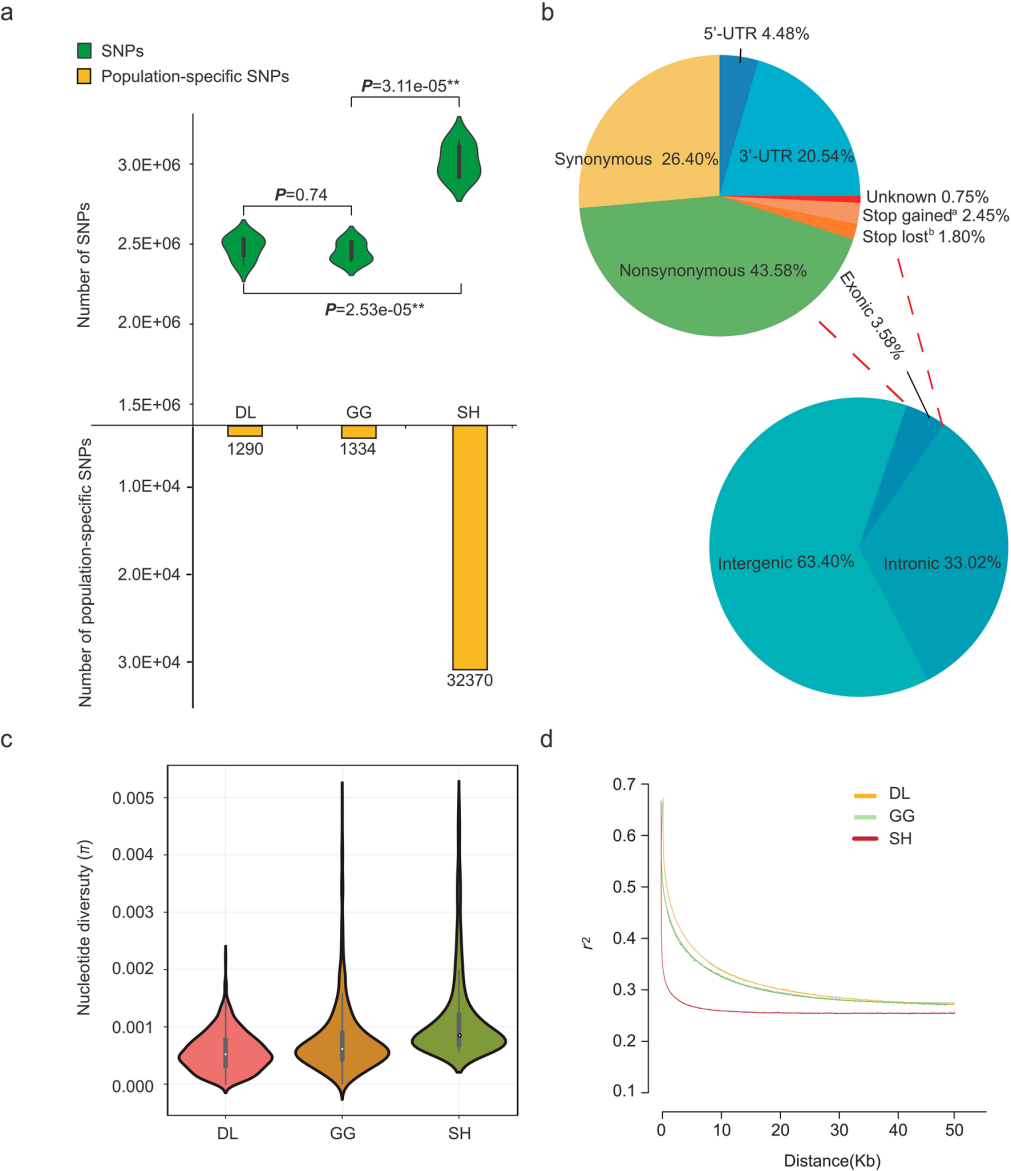
SNP identification and nucleotide diversity in Amur ide ecotypes. (**a**) The number of SNPs (positive y-axis) and population-specific SNPs (negative y-axis) identified in each population. (**b**) Functional classification of the candidate SNPs. (^a^Stop-gained: resulting in a premature stop codon in the coding sequence. ^b^Stop-lost: resulting in an elongated gene product because of stop codon loss.) (**c**) Violin plots of nucleotide diversity (*π*) for each population in 10-kb windows with10-kb steps. (**d**) LD decay estimated across the studied populations.

In this study, 679.82 Gb of clean data were collected, generating 48.56 Gb for each individual sample with 52.87-fold depth and 81.9% mapping rate on average to the reference genome of Amur ide (Supplementary Table S1). A total of 38,091,163 SNPs of high quality were obtained, including 12,610,411 in DL, 10,021,295 in GG, and 15,459,457 in SH. The SH population had the largest number of SNPs, and the difference was significant compared to the DL (two-tailed t-test, *P*_SNP(SH/DL)_ = 2.53e−05**) and GG (*P*_SNP(SH/GG)_ = 3.11e−05**) populations; while the two lake forms had no obvious differences in the number of SNPs (*P*_SNP (DL/GG)_ = 0.74) (Fig. 2a). In addition, we detected 1290 population-specific SNPs in DL, 1334 in GG and 32,370 in SH, and the types of SNP substitutions in each population were counted (Fig. 2a). The largest numbers of substitution mutations were G > A in the DL and GG populations and T > C in the SH population (Supplementary Table S2). With annotation, we identified 7,718,559 SNPs, among which 4,893,256 are intergenic (63.40%), 2,548,902 are intronic (33.025%), and 276,401 are exonic (3.58%). Subsequently, SNPs in exonic regions were analyzed in detail, we identified 76,830 synonymous (26.40%), 126,782 nonsynonymous (43.58%), 59,763 3′UTR (20.54%), 13,026 5′UTR (4.48%), 5233 stop-lost (1.80%), 7122 stop-gained (2.45%) and 2183 unknown (0.75%) sites (Table 1, Fig. 2b).

**Table 1:** Summary of SNP statistical information in Amur ide ecotypes.

Identified SNPs	DL	GG	SH	Total
Total SNPs	12,610,411	10,021,295	15,459,457	38,091,163
Annotated SNPs	6,011,522	5,897,735	6,396,784	7,718,559
Intergenic	3,693,576	3,504,953	3,736,659	4,893,256
Genic	2,317,946	2,392,782	2,660,125	2,825,303
Intronic	2,141,080	2,220,567	2,465,911	2,548,902
Exonic	176,866	172,215	194,214	276,401
3′-UTR	38,346	36,976	31,976	59,763
5′-UTR	9,326	9,287	7,382	13,026
Synonymous	44,324	43,329	54,844	76,830
Nonsynonymous	84,870	82,623	100,012	126,782
Stop gained	6328	6069	7033	7,122
Stop lost	4703	4481	5089	5,233
Unknown	1782	1857	2016	2,183

As Fig. 2c,d illustrated, SH had the highest nucleotide diversity and relatively fastest LD decay compared to DL and GG; GG had higher nucleotide diversity and relatively faster LD decay than those of DL; and DL had the lowest nucleotide diversity and the slowest LD level compared to the other two freshwater forms.

### Population structure

**Figure 3:**
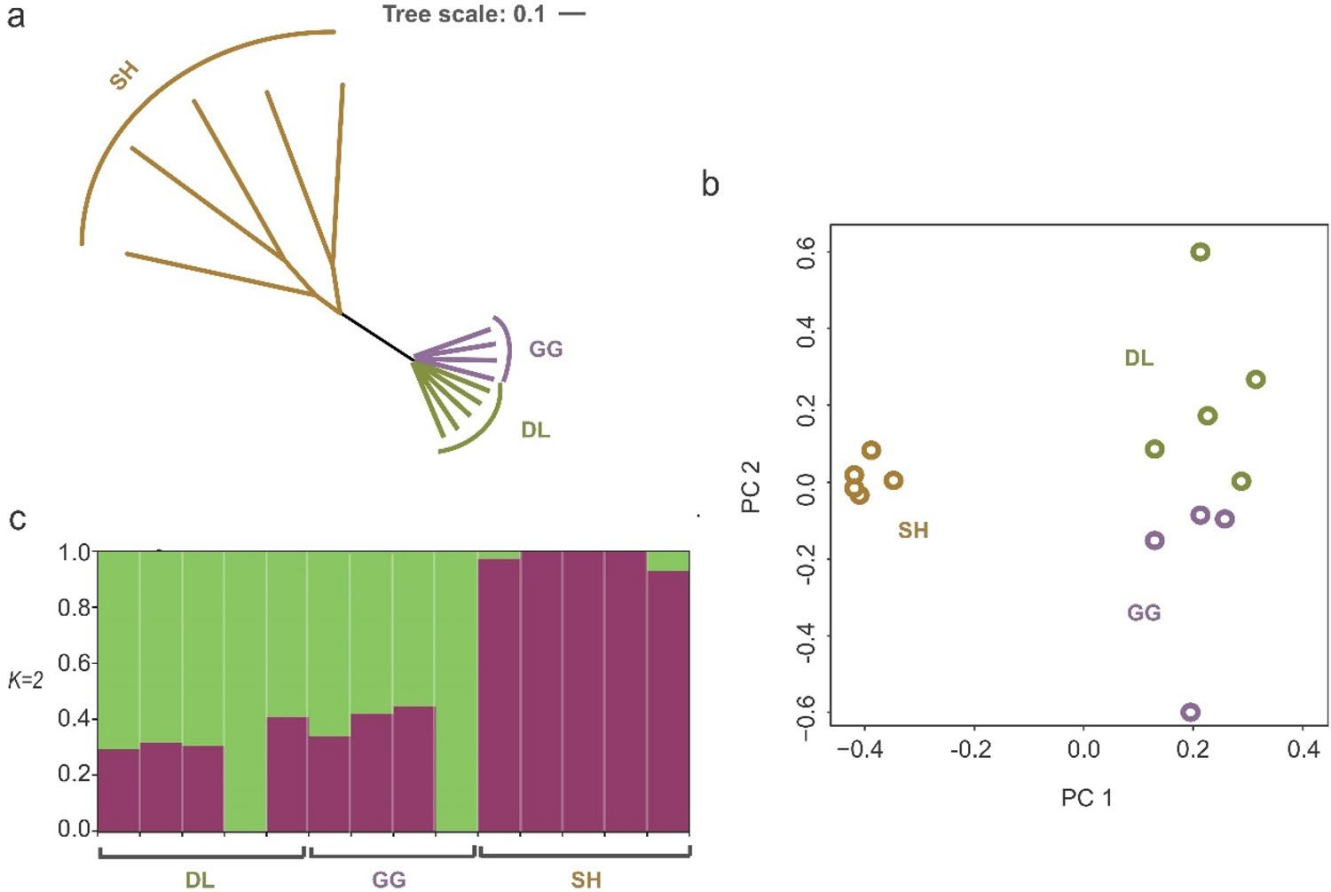
Population structure in Amur ide ecotypes. (**a**) Maximum-likelihood phylogenetic tree. (**b**) Principal component analysis, PC 1 against PC 2. (**c**) Population structure. Colors in each fragment represent the proportion of *K* = 2 ancestral populations assigned to individual genomes.

After pruning 7,718,559 annotated SNPs for LD analysis (*r*^2^ > 0.4), approximate 19,760 SNPs in coding regions were extracted from the filtered 427,051 SNPs to examine the population structure and genetic relationships of the three populations. The ML tree showed that the two lake forms of DL and GG clustered together with small genetic distances (genetic distance < 0.001), whereas the SH population clustered into a single group, which showed a higher level of diversity and a larger genetic distance (genetic distance = 0.17 on average) than those of the DL and GG populations (Fig. 3a). This result was further supported by PCA, which showed that the SH population was more differentiated than the DL and GG populations (Fig. 3b). Furthermore, population structure analysis results based on STRUCTURE were also consistent with the ML tree and PCA results. When setting a cluster with *K* = 2, all populations converged to two clusters with the highest average likelihood value. The SH population exhibited fewer admixed and diversified genetic components than the other two populations (Fig. 3c).

### Detection of selective candidate loci and genes associated with alkaline adaptation

**Figure 4:**
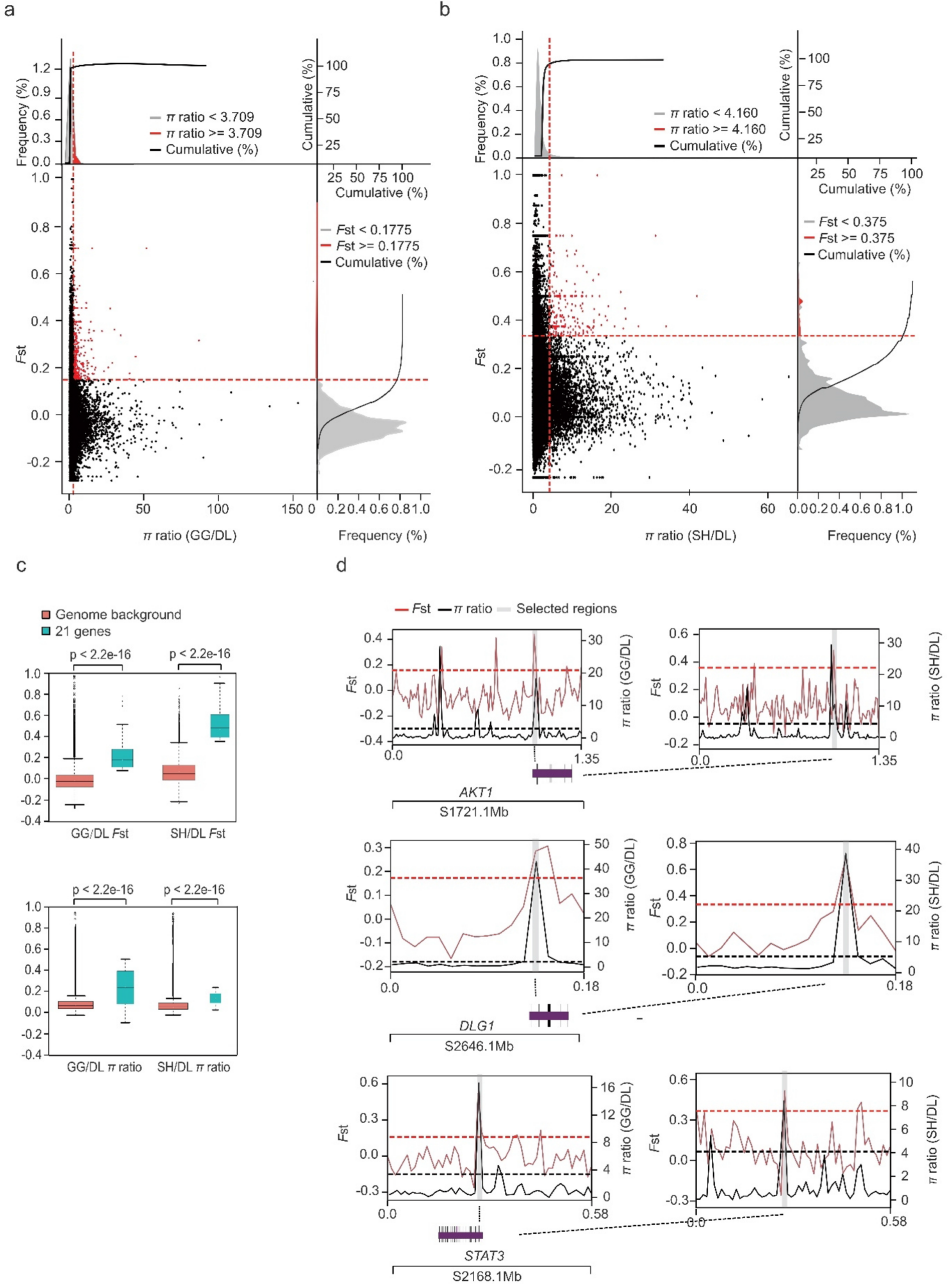
Genomic regions under selective sweeps. (**a**,**b**) Distribution of the *F*st and *π* ratio values, calculated in 10-kb windows with 10-kb sliding steps. Red data points were identified as selective sweeps that passed the thresholds of *π* ratio (the top 5% of the empirical distribution of *π* ratio, *π* ratio >= 3.709 in GG/DL and *π* ratio >= 4.160 in SH/DL) and *F*st (the top 5% of the empirical distribution of *F*st, *F*st >= 0.1775 in GG/DL and *F*st >= 0.375 in SH/DL). (**a**) The *F*st and *π* ratio of GG/DL. (**b**) The *F*st and *π* ratio of SH/DL. (**c**) Box plot of *π* ratio and *F*st values for twenty-one genes versus the whole genome. (**d**) Representative common genes of two pairwise groups with strong selective sweep signals. Genome annotations are shown at the bottom, and black bars represent coding sequences.

Genome-wide annotated SNPs were utilized to calculate *F*st and *π* ratio values of the two pairwise groups, which was performed with 10-kb window size and 10-kb step size. All windows containing less than 10 SNP sites were removed from the analysis. First, genomic loci with significantly high *F*st values (*P*_*F*st top 5% *vs.* 10-kb regions_ < 2.2e−16**) (0.1775 in GG/DL, 3258 windows and 0.375 in SH/DL, 3255 windows) and *π* ratios (*P*_*π* ratios top 5% *vs.* 10-kb regions_ < 2.2e−16**) (3.709 in GG/DL, 3229 windows and 4.160 in SH/DL, 3259 windows) were identified as highly divergent loci (Fig. 4a and Fig. 4b).Then, 367 common loci containing 242 genes in GG/DL and 447 common loci containing 325 genes in SH*/*DL shared by both *F*st and *π* ratio were detected. Finally, 51 common loci shared by two pairwise groups (GG*/*DL and SH*/*DL) were determined as candidate loci under positive selection of alkaline adaptation, with 21 genes being annotated (Table 2) (Supplementary Table S3). The strongest selective sweep signals were detected by comparing genomic regions under selective sweeps with the genome background, as shown by a box plot of the absolute difference in the *F*st and *π* ratio (Fig. 4c). Among these selected genes, many are involved in immune responses and hypoxia-related pathways, including *AKT1* (AKT serine/threonine kinase 1), *STAT3* (signal transducer and activator of transcription 3), and *DLG1* (discs large MAGUK scaffold protein 1) (Fig. 4d), of which *AKT1* and *STAT3 *are enriched in the “Toll-like receptors signaling pathway” and “HIF-1 signaling pathway” and *DLG1* in the “mitogen-activated protein kinase (MAPK) signaling pathway”.

**Table 2:** Genes located in selective loci shared by two pairwise groups (GG/DL and SH/DL).

Genes	Description	*F*st (GG/DL)	*π* ratio (GG/DL)	*F*st (SH/DL)	*π* ratio (SH/DL)
*PLEKHA7*	Pleckstrin homology domain-containing family A member 7	0.335477	9.321419	0.431818	11.599983
*GLI4*	Zinc finger protein	0.201935	20.714200	0.916667	16.222200
*CDHR1*	Cadherin-related family member 1	0.272943	9.241048	0.75	17.499972
*VIPR1*	Vasoactive intestinal polypeptide receptor 1	0.314159	33.348052	0.5	12.312491
*FTSJ3*	pre-rRNA processing protein	0.294009	6.040045	0.510054	4.725282
*TRAF6*	TNF receptor-associated factor 6	0.255486	7.457142	0.642857	4.679996
*SEC22B*	Vesicle-trafficking protein	0.291135	28.426183	0.553922	12.874990
*COL9A1*	Collagen alpha-1(IX) chain (precursor)	0.452055	9.508937	0.5	11.958344
*LMBRD1*	Lysosomal cobalamin transport escort protein	0.452055	9.508937	0.5	11.958345
*AKT1*	RAC-alpha serine/threonine-protein kinase	0.452055	18.950824	0.5	9.500012
*PSME4B*	Proteasome activator complex subunit 4B	0.452055	30.0000183	0.5	7.625011
*BRINP1*	BMP/retinoic acid-inducible neural-specific protein 1	0.182471	9.793534	0.394737	7.000004
*STAT3*	Signal transducer and activator of transcription 3	0.529966	17.099986	0.537879	8.679989
*RERG1*	Ras-related and estrogen-regulated growth inhibitor-like protein	0.199437	7.232139	0.416667	4.312485
*ARHGAP21b*	Rho GTPase-activating protein 21-B	0.256755	45.000000	0.482143	32.111100
*CTSL*	Cathepsin L1 light chain	0.382498	12.507772	0.434524	10.434778
*GNAI1*	Guanine nucleotide-binding protein G(i) subunit alpha-1	0.255014	20.390599	0.573438	16.437467
*PLCXD1*	PI-PLC X domain-containing protein 1	0.232040	4.620538	0.475	13.749986
*GSG11*	Germ cell-specific gene 1-like protein	0.341812	101.964499	1	18.000000
*DLG1*	Disks large homolog 1	0.250647	43.392850	0.675	37.777800
*CEP83*	Centrosomal protein of 83 kD	0.194631	12.857145	0.5	11.291670

### Enrichment analysis of PSGs and intersection genes associated with alkaline adaptation

**Figure 5:**
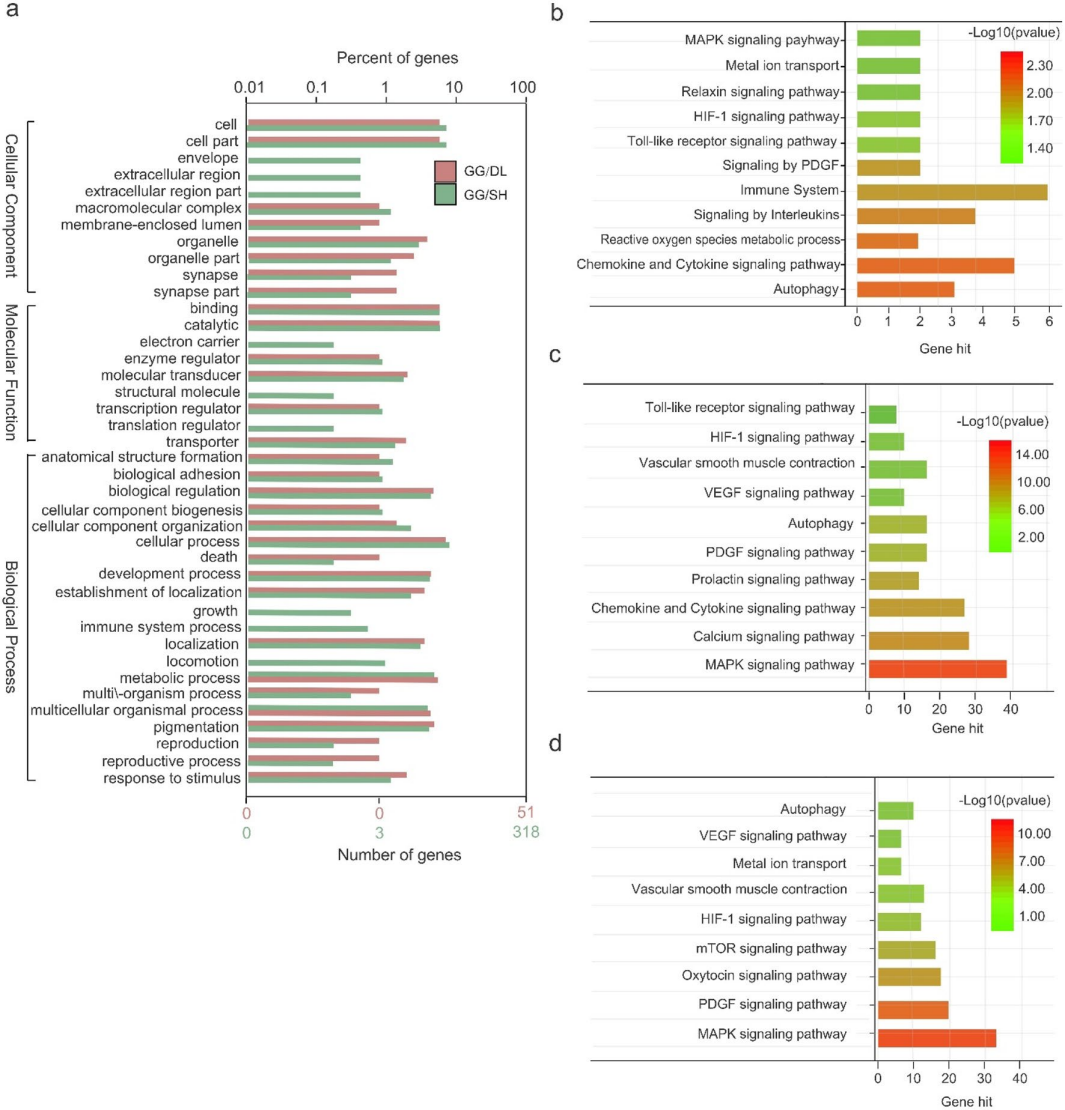
Enrichment analysis of candidate genes. (**a**) GO category analysis of genes using pairwise analysis. (**b**) KEGG category analysis of PSGs in the DL population. (**c**) KEGG category analysis of genes in stop-lost SNPs that are similar to PSGs. (**d**) KEGG category analysis of genes in stop-gained SNPs that are similar to PSGs.

WEGO revealed that the top-ranked GO terms from each pairwise group shared by both the highest *F*st and *π* ratio are related to local adaptations involving a range of biological processes, including biological regulation, cellular process, developmental process, metabolic process, pigmentation and response to stimulus (Fig. 5a). Further KEGG analysis showed that PSGs in the DL population cluster into several biological pathways, including autophagy, regulation of hypoxia-inducible factor (HIF) by oxygen, signaling by platelet-derived growth factor (PDGF), MAPK, relaxin signaling pathway, and metal ion transport (Fig. 5b) (Supplementary Table S4). Interestingly, some enrichment pathways that overlap with PSGs in the DL population were also found in gene enrichment analyses of stop-lost and stop-gained SNPs, such as the MAPK signaling pathway, chemokine and cytokine signaling pathway, PDGF signaling pathway, autophagy, HIF-1 signaling pathway, Toll-like receptor signaling pathway, and metal ion transport (Fig. 5c,d) (Supplementary Tables S5 and S6).

### Selective pressure analysis for candidate intersection genes associated with alkaline adaptation

**Figure 6:**
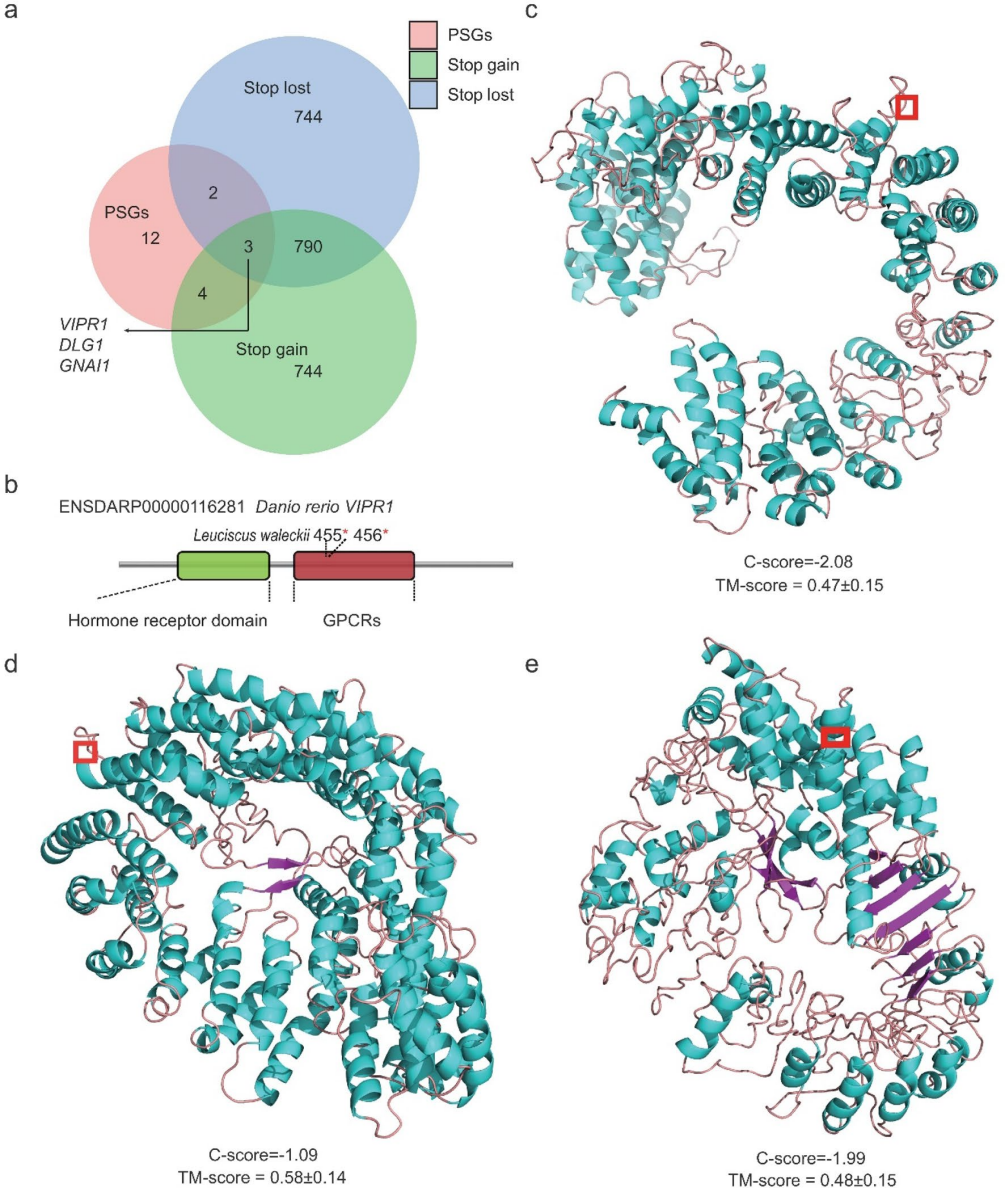
Target genes under selective pressure and differential VIPR1 protein structure in Amur ide ecotypes. (**a**) Venn diagram showing unique and overlapping genes among the PSGs and genes with stop-lost and stop-gained SNPs in DL, GG, and SH. (**b**) Structural analysis of the two positively selected sites of VIPR1. The protein coordinate is based on Ensembl ID ENSDARP00000116281. The lower panel shows the Pfam domains of the protein. The red stars indicate two sites (455th and 456th) that are essential for ligand binding. (**c**–**e**) Spatial distribution of positively selected sites in the 3D structure for Amur ide VIPR1. Three-dimensional views of VIPR1 proteins in DL (**c**), GG (**d**) and SH (**e**) highlight positively selected sites (455th and 456th) colored in the red rectangle frame.

The CodeML, MEME and FEL methods were used to detect the selective pressure of intersection genes among the PSGs and genes with stop-lost and stop-gained SNPs, and three genes, including *VIPR1*, *DLG1*, and *GNAI1*, were identified as being under selection (Fig. 6a). However, in addition to *VIPR1*, the site-based methods did not identify codons in *DLG1* and *GNAI1* under positive selective pressure. For *VIPR1*, positive selection of the 455th and 456th codons was found using the EasyCodeML and MEME methods, and positive selection of the 112th and 139th codons were detected in the MEME and FEL methods (Supplementary Tables S7 and S8). Among the four sites, only two (455th and 456th) are located in a well-defined protein domain (G protein-coupled receptors, GPCRs) (Fig. 6b). In addition, we examined the predicted 3D structures of VIPR1 in DL, GG and SH. The level of C-score confidence for three ecotypes were − 2.08, − 1.09, and − 1.99, respectively, indicating that the structures were constructed with high accuracy. The structural similarity and accuracy of the models were further checked using the TM-score, which showed all populations with TM-scores around 0.5 (0.47 ± 0.15, 0.58 ± 0.14, 0.48 ± 0.15, respectively), indicating that the modelled structure is of good quality. Interestingly, only the protein structure in DL has a wide and deep intracellular cavity in the core of VIPR1, which presumably forms a part of the peptide-binding site (Fig. 6c–e).

## Discussion

### Effects of sample size

The number of samples and molecular markers are two important parameters to evaluate genetic diversity and differentiation of pupations. However, an increasing body of evidence showed that small sample sizes across thousands of SNPs can be highly informative for studying the genetic differentiation and relationships of populations^11–15^. Specifically, Nazareno et al.^14^ suggested that even two samples per population are adequate when ≥ 1500 SNPs are used. Furthermore, a study by Patterson et al.^11^ showed that approximately 10 individuals per population and ~1000 SNPs will be enough if the true *F*st between two populations is 0.01. In the present study, despite only four to five samplers per population were used due to samples uneasy to obtained, a total of 19,760 SNPs were used for population structure analyses; combining to our previous results, the *F*st values were 0.0949–0.1185 between Amur populations (including SH) and Dali populations (including DL and GG) using microsatellite markers^2^. Thus, the sample size used in this study is well within what is considered.

### Nucleotide diversity and population structure

In this study, three parameters of SNP numbers, nucleotide diversity (*π*) and LD decay (*r*^2^) were used to evaluate nucleotide diversity of each population. According to our results, the freshwater form of SH had the largest number of SNPs, highest nucleotide diversity and relatively fastest LD decay, demonstrating that SH has increased levels of nucleotide diversity compared to DL and GG^3,16^. Relatively, the alkali form of DL had the lowest nucleotide diversity based on the values of the lowest nucleotide diversity and relatively slowest LD decay, which was mainly caused by increased inbreeding, limited gene flow and local adaptation inhabited in a lake without drainage as Chang et al.^2^ presumed before. Despite the freshwater form of GG likely experienced similar genetic events (e.g., genetic drift, bottleneck events, and inbreeding) as those that occurred in the DL population, it had moderate nucleotide diversity based on the values of higher nucleotide diversity and relatively faster LD decay compared to DL, the single direction of gene flow from DL to GG may explain for this based on the data reported by Chang et al.^2^

A clear population structure was found between the SH and the two lake populations of DL and GG (Fig. 3a–c). This is compatible with the geological evidence that the SH population was separated from populations from the Dali Basin and evolved into an independent population due to geographical isolation^2^. Furthermore, the two lake forms of the DL and GG were assigned to the same presumed population due to the similar genetic background^2^. However, the subsequent selective sweep analysis in our study identified some loci and genes associated with local adaptation in the alkaline environment of the DL population, implying that both geographical isolation and local adaptation caused the population divergence of Amur ide.

### Screening for genes associated with alkaline adaptation

Despite a few studies have found genes associated with alkaline adaptation by comparing Amur ide ecotypes using selective sweeps, the differences of spatial background were no consideration. Xu et al.^8^ firstly reported the genes associated with alkaline adaptation by comparing DL and GG based on transcriptomic data without considering their genetic exchange; subsequently, Xu et al.^9^ identified genes associated with alkaline adaptation by comparing DL and its ancestral freshwater form from Amur river based on genomic data without considering spatial background differences (geographical isolation and water system like lake *vs*. river). Therefore, to eliminate spatial background differences interference (geographical isolation, water system, genetic exchange) and focus on contrasting environments alone (alkaline water and freshwater), we made two sets of selective sweep analyses (DL *vs.* GG and DL *vs.* SH) and took the intersection between the gene lists generated by each pairwise group identified regions, and identified genes related to alkaline adaptation with high credibility. Finally, a total of 51 genomic loci with 21 candidate genes were detected. We used GO and KEGG enrichment analyses to identify biological pathways overrepresented with these genes, and a range of biological processes related to immune responses, blood vessel development, and osmotic regulation were found, demonstrating that genomic scanning is a reasonable and effective way to determine genomic signatures of local adaptation. This also explains the reason for the reduced diversity of the DL population from the perspective of local adaptation^7^.

Stop-lost and stop-gained SNPs are nonsense variants that result in truncated or incomplete gene products^17^. To obtain comprehensive evidence of local adaptation, we assessed whether stop-lost and stop-gained SNPs are enriched in specific gene functions with respect to biological processes. Interestingly, a multitude of biological processes were overrepresented with genes containing stop-gained variants. Among them, biological processes related to local adaptation are of great interest. More importantly, most of them overlap with the biological pathways of PSGs, indicating that stop-lost and stop-gained SNPs may also have profound effects on the stress response to extremely alkaline environments.

### Selective candidate genes with osmoregulatory regulation

Recent studies have shown that fish can sense changes in osmotic pressure in the external environment, and the pathways related to regulating osmotic pressure are transformed by sensory stimuli, thereby triggering many specific changes^18,19^. Phospholipase C (PLC) and MAPK signaling pathways are involved in osmotic pressure signaling in tilapia (*Oreochromis mossambicus*), killifish (*Oryzias latipes*) and turbot (*Scophthalmus maxima*)^20,21^.

In this study, we identified some genes (*DLG1*, *VIPR1*, *AKT1*, and *GNAI1*) and biological pathways (MAPK signaling pathway, relaxin signaling pathway, and metal ion transport) associated with mandatory physiological and structural alterations of osmoregulatory tissues^20,22^. The function of osmotic regulation affects a fish’s responses to extreme alkali–saline environments, and the genes and signaling pathways involved in this process can change the osmotic regulation strategies, which reverse the osmotic gradient between plasma/extracellular fluids and alkali–saline environments and complete the ion conversion between secretion and absorption.

There is no doubt that retention of water in the intestine occurs in response to alkaline and saline environments. VIPR1 belongs to G protein-coupled receptor class B1. In addition to vasoactive intestinal peptide (VIP), it binds to pituitary adenylate cyclase-activating polypeptide (PACAP), which is the most highly conserved member of the VIP-secretin-glucagon peptide superfamily. VIPR1 contributes to a variety of physiological functions, such as affecting the memory and learning system, stress response, neural development, immunomodulation, and exocrine secretion^23–27^. More importantly, it has been reported that VIPR1 acts on intestinal smooth muscle relaxation in mammals and promotes the discharge of water and electrolytes in the digestive system^28,29^. A few studies have reported that PACAP can relax smooth muscle in the stargazer by inhibiting contractions stimulated by acetylcholine or potassium chloride^30^, and recent evidence based on cryo-electron microscopy (cryp-EM) has further confirmed that PACAP27 (one form of PACAP) is embedded in the cavity of the VIPR1 protein structure ^31^*.* In the present study, *VIPR1* is at the gene intersection among positive selection scanning, stop-gained, and stop-lost. Further 3D protein modeling showed that the opening of the hydrophobic receptor binding site in the cavity of VIPR1 is enlarged in the DL population, implying that VIPR1 in the DL population may be more effective at ligand binding than VIPR1 in the other two freshwater populations. Thus, we postulated that VIPR1 may play an important role in maintaining hydromineral balance during alkaline adaptation, which promotes smooth muscle relaxant actions by combining with PACAP in the digestive tract of the DL population.

### Selective candidate genes associated with inflammation and immune responses

Recently, many studies have shown that some fish species living in harsh environments will stimulate many genes related to the immune system to fight against unfavorable habitats in the long term. Using comparative transcriptomics, Liang et al.^32^ found that a number of immune-related genes were triggered in the spleen of Amur carp (*Cyprinus carpio haematopterus*) at cooling temperatures. Tong et al.^33^ also reported that innate immune-related pathways were the most highly enriched in naked carp (*Gymnocypris przewalskii*), an alkali-saline-tolerant species inhabiting Lake Qinghai of China, to cope with the harsh living environment and pathogens (“white spot disease”).

In this study, we found that several genes (e.g., *DLG1*, *BRINP1*, *CTSL, TRAF6, AKT1, STAT3, GNAI1, SEC22b,* and *PSME4b*) and biological pathways (e.g., autophagy, Toll-like receptor signaling pathways, adaptive immune system, chemokine and cytokine signaling pathways, and signaling by interleukins) are associated with inflammation and immune responses, which might reflect the adaptation process of the DL population to the extreme alkali–saline environment. Apoptosis and tissue injury caused by the extreme environment cause damaged cells to release molecules that act as endogenous signals for the activation of inflammasome pathways and affect immune responses^18^. The immune system promotes the adaptation of the extreme alkali–saline environment by enhancing a variety of cytoprotective responses, thereby providing defense against inflammation and tissue damage caused by the environment. Moreover, the organism will activate the hypoxia signal pathway under hypoxic conditions and stimulate T cell differentiation and cytokine synthesis, thereby inhibiting the accumulation of inflammatory factors in the cell, repairing damaged blood vessels, and restoring the organism's balance^34,35^.

### Selective candidate genes associated with cardiorespiratory development

Oxygen is the key factor for maintaining normal life activity and metabolism in fish. Some biotic and abiotic factors, such as temperature, ion concentration, pH, microorganisms, and algae, will change dissolved oxygen levels^36–38^, and some studies have indicated that the oxygen dissolution rate decreases with increasing water ion concentration^39–42^.

Considering the harsh environment with high alkalinity in Lake Dali Nor, inadequately dissolved oxygen is also a factor threatening the survival of the DL population^35^. In this study, we identified some genes (e.g., *TRAF6*, *PSME4b*, *STAT3*, *AKT1*, and *COL9A1*) and biological pathways (e.g., MAPK signaling pathway, HIF-1 signaling pathway, reactive oxygen species metabolic process, and signaling by PDGF) associated with cardiorespiratory development, indicating that the differentiation and development of blood vessels and cardiomyocytes play important roles in the DL population while living in a hypoxic environment^43–45^. Genes and signaling pathways involved in cardiorespiratory function can compensate for adverse effects caused by insufficient levels of dissolved oxygen in water, improve the efficiency of gas exchange in organisms, and maintain the stability of oxygen concentration^46^.

It is worth noting that several studies have focused on exploring *HIF* family genes related to hypoxia adaptation in fish. Eurasian perch (*Perca fluviatilis*) can upregulate expression of *HIF-1a* in the brain and liver under acute hypoxia conditions, while *HIF-1a* expression can change significantly in the muscle under chronic hypoxia conditions^47^. Indian catfish (*Clarias batrachus*) significantly upregulate three *HIF-α* (*HIF-1α*, *HIF-2α*, and *HIF-3α*) transcripts in the brain, liver, and head kidney under short-term hypoxia exposure; under long-term hypoxia exposure, *HIF-1α* in the spleen and *HIF-2α* in the muscle are significantly upregulated, and *HIF-3α* is downregulated in the head kidney^48^. Using genetic linkage analysis, Wang et al.^49^ identified *HIF-3α* as involved in alkaline adaptation of the DL population by suppressing gene expression in the gills. These findings implied that HIF family genes act as important modulators regulating angiogenesis. In addition, the VEGF signaling pathway regulates angiogenesis through increased expression of *HIF-1*^45^, and interactions between the MAPK and VEGF signaling pathways can increase angiogenesis^50^.

## Materials and methods

### Ethics statement

In this study, all experiments involving the handling and treatment of fish were approved by the Animal Care and Use committee of Heilongjiang River Fisheries Research Institute of Chinese Academy of Fishery Sciences (HRFRI). The methods were carried out in accordance with approved guidelines. Before the blood samples were collected, all the fishes were euthanized in MS222 solution. In addition, we have followed the ARRIVE guidelines (http://www.nc3rs.org.uk/arrive-guidelines)^51^.

### Sampling, DNA extraction, and resequencing

Three populations of Amur ide collected from Lake Dali Nor (DL), Lake Ganggeng Nor (GG) and the Songhua River (SH) were used in this study, including five individuals from DL, four individuals from GG, and five individuals from SH. Genomic DNA was extracted from each blood sample using a DNeasy Blood and Tissue Kit (Qiagen, Germany). The DNA concentration and integrity were evaluated using a NanoDrop 8000 (NanoDrop Technologies, USA) and 1% agarose gel electrophoresis. DNA libraries were prepared with greater than 1 μg of starting total DNA following Illumina protocols (Illumina Inc., USA), and then whole genome resequencing was completed on an Illumina HiSeq 4000 (Illumina Inc., USA) sequencing platform with a paired-end 150-bp strategy. The adaptors and low-quality bases (q < 20) were filtered out to obtain a set of clean paired reads by the FASTX-Toolkit (version 0.0.13) (http://hannonlab.cshl.edu/fastx_toolkit/).

### SNP calling and annotation

The cleaned paired-end reads were mapped to the Amur ide reference genome (NCBI Accession Number GCA_900092035.1, Xu, et al. ^9^) using Bowtie2 (version 2.3.5)^52^. SNPs were called using the ‘mpileup’ command in SAMtools (version 1.9)^53^ and saved as bcf files (.bcf). All bcf files (.bcf) were then converted to vcf files (.vcf) with bcftools (version 1.9). In addition, we used bcftools to filter the vcf files. Unreliable SNPs that exhibited the following features were filtered out using bcftools: (1) coverage depth < 10 or > 1000; (2) root mean square (RMS) mapping quality < 20; and (3) read quality value < 20. Violin plots of the number of SNPs in each population were made with ggplot2^54^ in the R package, and the significance levels were analyzed with a two-tailed Students t-test using R (version 3.6.3)^55^. Subsequently, we annotated and predicted the effects of the SNPs using snpEff (version 4.3)^56^. We constructed the necessary snpEff databases for Amur ide using GFF3 and FASTA files from reference assemblies of Xu et al.^9^. First, we obtained the publicly available Amur ide genome and annotation files, and renamed them as “genes.fa” and “genes.gff3”, respectively. Next, these two files were installed into the subfolder we created, the pre-existing “data” subfolder in the snpEff installation. We added a FASTA-formatted file containing the Amur ide genome reference sequences into the pre-existing “genome” subfolder in the above “data” folder, and genome annotation into the pre-existing "amur_ide" subfolder. We then added a new genome entry in the “snpEff.config” file in the snpEff directory: “amur_ide.genome: amur_ide”. Finally, we used the snpEff build command with parameters “-gff3 -v” to construct a custom snpEff database for Amur ide. Predicted effects of the detected SNPs were computed by the snpEff eff command with parameter option “-c”. The output file was then post-processed using a custom Python (version 3.6) script to isolate each component. Furthermore, population-specific SNPs were identified using the SnpSift component (version 4.1)^57^ in the snpEff java package.

### Nucleotide diversity

We investigated the nucleotide diversity (*π*) for each population using VCFtools (version 0.1.13)^58^ in 10-kb non-overlapping windows (–window-pi 10000 –window-pi-step 10000). Prior to the LD decay analyses, the resulting filtered vcf files were converted to PLINK (.ped and .map) format file using VCFtools. The parameter *r*^2^ for LD was calculated using PLINK with the parameters (–ld-window -*r*^2^ 0 –ld-window 99999 –ld-window-kb 50). The average *r*^2^ value was calculated for each length of distance and plotted against the physical distances of SNPs in units of kb. The corresponding figures were drawn by an R script^55^.

### Population structure

All annotated SNPs were pruned using the indep-pairwise option (plink –file data –indep-pairwise 50 10 0.4) in PLINK (version 1.07)^59^ to avoid the strong influence of linked SNP clusters in relatedness analysis. We then extracted SNPs within the coding region in the filtered dataset for population structure and genetic relationships research. A maximum-likelihood (ML) tree was constructed based on the SNPs from the coding regions by using PhyML (HKY85 model) (version 3.1)^60^ and was plotted with iTOL (version 4.4.2). GCTA (version 1.91.1)^61^ was applied for principal component analysis (PCA). The Bayesian clustering program STRUCTURE (version 2.3.4)^62^ was used to analyze the distribution of SNPs among populations with 2000 iterations. StructurePlot (version 2.0)^63^ was performed to display the individual clusters of the estimated population structure.

### Detection of selective signatures

To detect selection signatures associated with alkaline adaptation, we combined two approaches to select the positively selected genes (PSGs), including *F*st and *π* ratio (*π*_freshwater_/*π*_alkaline water_) of the freshwater forms of GG and SH to alkaline form of DL. First, we calculated the *π* ratio for DL, GG, and SH using VCFtools with a non-overlapping sliding window approach (10 kb windows with 10-kb stepwise distance, -window -pi 10000). The size and steps of the sliding window were based on empirical evaluation and referred to the practice of Xu et al.^9^. We separately estimated the *π* ratio (*π*_GG/*π*_DL and *π*_SH/*π*_DL). Secondly, we calculated the genome-wide distribution of *F*st values using VCFtools with the same window size and stepwise distance (-fst -window -size 10000 -weir -fst -pop). Thirdly, loci from each pairwise group (GG/DL and SH*/*DL) with high *F*st and *π* ratio values (corresponding to a top 5% level) were identified as selected loci. For each pairwise group, we compared *F*st and *π* ratio values of top 5% SNP loci with those of 10-kb region by Students t-tests in R^55^ to determine significance. Finally, the selected loci shared by two pairwise groups were identified as highly divergent and PSGs were annotated. Furthermore, JBrowser (version 1.12.3)^64^ was used for the visualization of identified PSGs.

### Enrichment analysis

Genes at selected loci from each pairwise group were classified into the GO database^65^ using WEGO (http://wego.genomics.org.cn/) and were annotated using KOBAS 3.0 (http://kobas.cbi.pku.edu.cn/) for the subsequent pathway analysis with the Kyoto Encyclopedia of Genes and Genomes (KEGG)^66–68^ and PANTHER^69^. Considering the drastic impact of selective SNPs on phenotypes^17^, genes in stop-lost and stop-gained SNPs from snpEff were also extracted and uploaded to KOBAS 3.0 for enrichment pathway analysis. *P* values were corrected by the False Discovery Rate (FDR) method of Benjamini–Hochberg and a significance threshold of FDR-corrected *P* < 0.05 was applied.

### Selection pressure analysis for candidate intersection genes

We used the intersection among the PSGs and genes with stop-lost and stop-gained SNPs to conduct selection pressure analysis. First, the transcript sequences from the three Amur ide populations were retrieved. Then, the gene orthologs of six other representative outgroup fish species were obtained from GenBank (http://www.ncbi.nlm.nih.gov/genbank/); the accession numbers of the sequences were as follow: zebrafish (*Danio rerio*), XM_005162698; Nile tilapia (*Oreochromis niloticus*), XM_003439191; Mexican tetra (*Astyanax mexicanus*), XM_007249044; turquoise killifish (*Nothobranchius furzeri*), XM_015955164; Indian medaka (*Oryzias melastigma*), XM_024280491; and goldfish (*Carassius auratus*), XM_026200457. Next, these sequences were aligned using the ClustalW program (version 2.1)^70^, followed by manual adjustment. Finally, a phylogenetic tree was constructed using the ML method in the Mega X software^71^ with 1000 bootstrap replications. These output files were used in subsequent selective pressure analysis.

Three methods were used to perform selective pressure analysis. One was the CodeML method^72^ implemented in the EasyCodeML software (version 1.21)^73^. It was performed to calculate the non­synonymous to synonymous substitution rate ratio (*ω* = *dN/dS*), where *ω* = 1, *ω* < 1 and *ω* > 1 correspond to neutral, purifying and positive selection, respectively^74^. A site-specific model was used to identify the variation of selective pressures on these genes, which allowed the *ω* ratio to vary among sites with a fixed *ω* ratio in all branches. Seven codon substitution models described as M0 (one-ratio), M1a (neutral), M2a (positive selection), M3 (discrete), M7 (β), M8 (β and *ω* > 1) and M8a (β and *ω* = 1) were investigated, and four different nested models (M0 *vs.* M3, M1a *vs.* M2a, M7 *vs.* M8 and M8a *vs.* M8) were calculated to detect positive selection on each site for candidate intersection genes^75–80^. The significance of differences between each two nested models was assessed using likelihood ratio tests (LRTs). Positively selected sites of genes with *p* < 0.05 in LRTs were further evaluated using Bayes Empirical Bayes (BEB)^80^ analysis with posterior probabilities ≥ 0.95. The other two methods were the mixed-effects model of evolution (MEME) and fixed effects likelihood (FEL) methods in the HyPhy package^81^ implemented on the Datamonkey server (http://www.datamonkey.org/). These methods were also used to infer the positively selected sites. Sites were considered candidates under positive selection when they met the following conditions: β +  > α, significant likelihood ratio test (*p* < 0.05) in MEME, and *p* < 0.05 in the FEL likelihood ratio test. Sites identified by at least two methods were regarded as robust candidate positive selection sites.

### Three-dimensional (3D) structure modeling of candidate intersection genes

To verify whether the positive selection sites obtained were located in important protein functional domains, the amino acid sequences of candidate genes from three Amur ide populations were aligned to corresponding zebrafish sequences to determine the equivalent positions of positively selected sites. Next, the Pfam webserver^82^ was used to determine whether the sites in zebrafish homologues have functional effects or are in proximity to functionally annotated sites. Then, the I-TASSER webserver (https://zhanglab.ccmb.med.umich.edu/I-TASSER/)^83,84^ was used to predict the 3D structure of the candidate genes and the structure was evaluated by two scoring function C-score and TM-score. For C-score, a good predicted model was obtained from a protein sequence when the estimated level of confidence was between [−5-2]. For TM-score, a model with TM-score > 0.5 indicates correct topology, and < 0.17 means the predicted structure with low accuracy^85^. Finally, the resulting model was visualized with PyMOL (http://pymol.sourceforge.net/).

## Conclusion

Amur ide provides an excellent model for understanding the genetic basis of alkaline adaptation evolutionarily from the genomic level. Here, in order to eliminated spatial background differences interference (geographical isolation, water system, genetic exchange) and fucus on contrasting environments alone (alkaline water and freshwater), we re-sequenced genomes of three populations of Amur ide inhabiting different environments and identified 21 functional genes related to alkaline adaptation using selective sweeps based on a larger number of SNPs. Enrichment analysis showed that these genes mainly involved in osmoregulatory regulation, inflammation and immune responses, and cardiorespiratory development, which probably played important roles for Amur ide during alkaline adaptation. Further experiments are required to establish cause-and-effect relationships between phenotype and genotype. In summary, this study provides useful data for clarifying the genetic mechanisms of alkaline adaptation of Amur ide in the future.

## Supplementary Information

Supplementary Information
