# Supplementary material for: Resequencing and SNP discovery of Amur ide (Leuciscus waleckii) provides insights into local adaptations to extreme environments
Source: Sci Rep. 2021 Mar 3;11:5064. doi: 10.1038/s41598-021-84652-5 (PMC7930030; doi:10.1038/s41598-021-84652-5)
Supplement: Supplementary file 1 — Supplementary Information [file 41598_2021_84652_MOESM1_ESM.docx]

**Resequencing and SNP Discovery of Amur ide (*Leucis*c*us waleckii)* Provides Insights into Local Adaptations to Extreme Environments**

Shuangyi Wang^1,2^, Youyi Kuang^1^, Liqun Liang^1^, Bo Sun^1^, Xuefei Zhao^1,3^, Limin Zhang^1^, Yumei Chang^1,*^

^1^National & Local United Engineering Laboratory of Freshwater Fish Breeding, Heilongjiang River Fisheries Research Institute, Chinese Academy of Fishery Sciences, Harbin 150070, China

^2^College of Fisheries and Life Science, Shanghai Ocean University, Shanghai 200000, China

^3^College of Wildlife and Protected Area, Northeast Forestry University, Harbin 150040, China

^*^Corresponding. changyumei@hrfri.ac.cn

**SUPPLEMENTARY INFORMATION**

Table of Contents

[Supplementary Tables 3](#_Toc63366533)

[Supplementary Table S1. Summary of sequencing data quality 3](#_Toc63366534)

[Supplementary Table S2. Specific substitution type of SNPs in each population 4](#_Toc63366535)

[Supplementary Table S3. Genes in the selected regions (the top 5% of the empirical *F*st and *π* distribution) 5](#_Toc63366536)

[Supplementary Table S4. Functional categories of PSGs in DL population 7](#_Toc63366537)

[Supplementary Table S5. Functional categories of genes in stop-lost 8](#_Toc63366538)

[Supplementary Table S6. Functional categories of genes in stop-gained 9](#_Toc63366539)

[Supplementary Table S7. Model based evolutionary analysis of positive selection by CodeML method 10](#_Toc63366540)

[Supplementary Table S8. Sites found to be under positive selection by MEME and FEL methods 11](#_Toc63366541)

# Supplementary Tables

## Supplementary Table S1. Summary of sequencing data quality

| Sample | Raw Reads | Clean Reads | Mapped reads | Mapping rate | Raw Base(G) | Clean Base(G) | Ave_fold^1^ |
| --- | --- | --- | --- | --- | --- | --- | --- |
| DL1 | 162,222,425 | 161,649,675 | 265,765,182 | 82.20% | 48.67 | 48.49 | 52.99 X |
| DL2 | 161,460,644 | 160,720,821 | 263,095,847 | 81.85% | 48.44 | 48.22 | 52.46 X |
| DL3 | 159,997,375 | 159,126,124 | 261,486,938 | 82.16% | 48 | 47.74 | 52.14 X |
| DL4 | 187,330,224 | 186,465,486 | 307,344,683 | 82.41% | 56.2 | 55.94 | 61.28 X |
| DL5 | 162,840,529 | 162,018,922 | 266,412,579 | 82.22% | 48.85 | 48.61 | 53.12 X |
| GG1- | 155,317,008 | 154,474,210 | 254,519,116 | 82.38% | 46.59 | 46.34 | 50.75 X |
| GG2 | 154,765,663 | 153,931,039 | 254,023,068 | 82.51% | 46.43 | 46.18 | 50.65 X |
| GG3 | 167,617,300 | 166,733,146 | 273,912,932 | 82.14% | 50.28 | 50.02 | 54.61 X |
| GG4 | 169,879,040 | 169,021,920 | 278,910,785 | 82.51% | 50.96 | 50.71 | 55.61 X |
| SH1 | 162,095,932 | 161,152,950 | 260,945,026 | 80.96% | 48.63 | 48.35 | 52.03 X |
| SH2 | 160,483,034 | 159,652,238 | 259,156,951 | 81.16% | 48.15 | 47.9 | 51.67 X |
| SH3 | 156,835,816 | 156,162,082 | 254,561,923 | 81.51% | 47.05 | 46.85 | 50.76 X |
| SH4 | 154,283,488 | 153,529,321 | 251,545,155 | 81.92% | 46.28 | 46.06 | 50.15 X |
| SH5 | 162,234,838 | 161,365,647 | 260,829,400 | 80.82% | 48.67 | 48.41 | 52.00 X |
| **Total** | 2,277,363,316 | 2,266,003,581 | 3,712,509,585 | X | 683.20 | 679.82 | X |
| **Average** | 162,668,808 | 161,857,398 | 265,179,256 | 81.90% | 48.80 | 48.56 | 52.87 |

^1^ Ave_Fold, average fold that was calculated as the average depth of coverage across the whole genome.

## Supplementary Table S2. Specific substitution type of SNPs in each population

| DL | SNP count | GG | SNP count | SH | SNP count |
| --- | --- | --- | --- | --- | --- |
| G > A | 786 | G > A | 577 | T > C | 17835 |
| C > T | 593 | C > T | 554 | C > T | 17289 |
| T > C | 466 | T > C | 391 | A > G | 16981 |
| A > T | 402 | T > A | 386 | G > A | 16766 |
| A > G | 373 | A > G | 346 | A > T | 9898 |
| G > T | 367 | G > T | 310 | T > A | 9793 |
| C > A | 341 | A > T | 305 | T > G | 9595 |
| T > G | 334 | C > A | 299 | A > C | 9423 |
| A > C | 320 | A > C | 263 | C > A | 9231 |
| T > A | 291 | T > G | 230 | G > T | 8833 |
| G > C | 184 | C > G | 172 | C > G | 4749 |
| C > G | 167 | G > C | 149 | G > C | 4575 |

## Supplementary Table S3. Genes in the selected regions (the top 5% of the empirical *F*st and *π* distribution)

| chr | start | end | *π* ratio（GG/DL） | mean F*st*（GG/DL） | π ratio(SH/DL) | mean F*st*（SH/DL） | gene id |
| --- | --- | --- | --- | --- | --- | --- | --- |
| 173 | 10001 | 20000 | 30.13393146 | 0.393939 | 26.0624766 | 0.5 | CAFS_LW_G_12642; |
| 116 | 610001 | 620000 | 9.321418535 | 0.335477 | 11.59998272 | 0.431818 | CAFS_LW_G_07893; |
| 269 | 130001 | 140000 | 4.691117475 | 0.256926 | 5.162158151 | 0.5 | CAFS_LW_G_19210; |
| 310 | 10001 | 20000 | 4.285711224 | 0.263288 | 6.619045283 | 0.375 |  |
| 411 | 10001 | 20000 | 10 | 0.18584 | 8 | 0.4375 |  |
| 445 | 80001 | 90000 | 20.7142999 | 0.201935 | 16.22220002 | 0.916667 | CAFS_LW_G_01312; |
| 151 | 500001 | 510000 | 9.241047807 | 0.272943 | 17.49997187 | 0.75 | CAFS_LW_G_09899; |
| 382 | 360001 | 370000 | 11.85267574 | 0.510021 | 18.18748673 | 0.5 |  |
| 287 | 750001 | 760000 | 33.34805205 | 0.314159 | 12.31249092 | 0.5 | CAFS_LW_G_19387; |
| 186 | 520001 | 530000 | 6.040045088 | 0.294009 | 4.725282115 | 0.510054 | CAFS_LW_G_14352; |
| 186 | 530001 | 540000 | 28.12496484 | 0.537636 | 27.62495957 | 0.425 | CAFS_LW_G_14352; |
|  |  |  |  |  |  |  | CAFS_LW_G_14411; |
| 696 | 40001 | 50000 | 45.89284928 | 0.200033 | 15.3333501 | 0.589286 | CAFS_LW_G_20476; |
| 555 | 700001 | 710000 | 7.515750006 | 0.226123 | 10.61763787 | 0.528846 | CAFS_LW_G_20277 |
|  |  |  |  |  |  |  | ;CAFS_LW_G_02056; |
| 716 | 440001 | 450000 | 21.09376308 | 0.194631 | 4.833340522 | 0.5 |  |
| 719 | 230001 | 240000 | 20.26785018 | 0.401268 | 10.55555 | 0.545455 |  |
| 1036 | 80001 | 90000 | 29.73202496 | 0.314159 | 23.31247411 | 0.411765 |  |
| 841 | 1080001 | 1090000 | 7.457142015 | 0.255486 | 4.679996248 | 0.642857 | CAFS_LW_G_04559; |
| 1052 | 220001 | 230000 | 23.57144992 | 0.278989 | 23.33335013 | 0.615385 |  |
| 1128 | 50001 | 60000 | 4.821425117 | 0.384191 | 5.142853478 | 0.75 |  |
| 1146 | 410001 | 420000 | 6.629447973 | 0.314159 | 10.31249649 | 0.75 |  |
| 1258 | 70001 | 80000 | 9.241047807 | 0.304625 | 22.8749618 | 0.65 |  |
| 1020 | 580001 | 590000 | 28.42618296 | 0.291135 | 12.87499021 | 0.553922 | CAFS_LW_G_06090; |
| 1025 | 660001 | 670000 | 13.29545158 | 0.72028 | 4.272728572 | 0.75 |  |
| 1409 | 110001 | 120000 | 9.508937184 | 0.452055 | 11.95834497 | 0.5 | CAFS_LW_G_08824; |
|  |  |  |  |  |  |  | CAFS_LW_G_08831; |
| 1378 | 620001 | 630000 | 5.197129351 | 0.315802 | 4.389612584 | 0.43787 |  |
| 1282 | 180001 | 190000 | 5.926346231 | 0.319241 | 5.249996127 | 0.408163 |  |
| 1413 | 1870001 | 1880000 | 24.64295318 | 0.194631 | 9.083330703 | 0.625 |  |
| 1643 | 100001 | 110000 | 22.14284995 | 0.22432 | 33.11110046 | 0.472222 |  |
| 1735 | 1 | 10000 | 5.714299984 | 0.393939 | 10.77779998 | 0.5 | CAFS_LW_G_21880; |
| 1851 | 60001 | 70000 | 4.821434602 | 0.72028 | 10 | 0.375 | CAFS_LW_G_11651; |
| 1721 | 960001 | 970000 | 18.95082437 | 0.452055 | 9.500012174 | 0.5 | CAFS_LW_G_10734; |
| 1502 | 1070001 | 1080000 | 6.562504108 | 0.452055 | 4.708334188 | 0.5 |  |
| 1976 | 200001 | 210000 | 30.0000183 | 0.452055 | 7.625010988 | 0.5 | CAFS_LW_G_12436; |
| 2169 | 110001 | 120000 | 22.60040637 | 0.39139 | 4.624981716 | 0.625 |  |
| 1891 | 330001 | 340000 | 15.66962734 | 0.314159 | 7.249997187 | 0.411765 | CAFS_LW_G_11923; |
| 1891 | 400001 | 410000 | 9.793534373 | 0.182471 | 7.000004207 | 0.394737 | CAFS_LW_G_11923; |
| 2168 | 280001 | 290000 | 17.0999863 | 0.529966 | 8.679989021 | 0.537879 | CAFS_LW_G_13637; |
| 2192 | 510001 | 520000 | 7.232137833 | 0.199437 | 4.312485233 | 0.416667 | CAFS_LW_G_22389; |
| 2327 | 160001 | 170000 | 10.34596514 | 0.314159 | 13.12499297 | 0.411765 |  |
| 2467 | 190001 | 200000 | 9.285700032 | 0.702317 | 19.22219983 | 0.5 |  |
| 2467 | 540001 | 550000 | 45.00000045 | 0.256755 | 32.1111003 | 0.482143 | CAFS_LW_G_15421; |
| 2567 | 1 | 10000 | 7.433034473 | 0.314159 | 7.999977472 | 0.75 | CAFS_LW_G_16651; |
| 2559 | 260001 | 270000 | 12.50777233 | 0.382498 | 10.4347784 | 0.434524 | CAFS_LW_G_16632; |
| 2563 | 230001 | 240000 | 12.4553375 | 0.314159 | 10.37499012 | 0.411765 | CAFS_LW_G_16650; |
| 2637 | 130001 | 140000 | 20.39059947 | 0.255014 | 16.43746701 | 0.573438 | CAFS_LW_G_17159; |
|  |  |  |  |  |  |  | CAFS_LW_G_17160; |
| 2709 | 90001 | 100000 | 4.620537972 | 0.23204 | 13.74998596 | 0.475 | CAFS_LW_G_17502; |
|  |  |  |  |  |  |  | CAFS_LW_G_17501; |
| 2621 | 160001 | 170000 | 101.964499 | 0.341812 | 17.99999986 | 1 | CAFS_LW_G_17051; |
| 2646 | 130001 | 140000 | 43.39285053 | 0.250647 | 37.77779952 | 0.675 | CAFS_LW_G_17212; |
| 2884 | 60001 | 70000 | 12.55100525 | 0.264163 | 9.142857728 | 0.375 | CAFS_LW_G_18037; |
| 2706 | 90001 | 100000 | 12.85714547 | 0.194631 | 11.29166955 | 0.5 | CAFS_LW_G_17516; |
| 3695 | 1 | 10000 | 3.816958634 | 0.410675 | 5.333328327 | 0.525 | CAFS_LW_G_18719; |

## Supplementary Table S4. Functional categories of PSGs in DL population

| pathway | p-value | (-log10pvalue) | gene |
| --- | --- | --- | --- |
| Autophagy | 0.005017 | 2.299555899 | *3(AKT1、CTSL、TRAF6)* |
| Chemokine and Cytokine signaling pathway | 0.006965 | 2.157078879 | *5(AKT1、STAT3、GNAI1、TRAF6、DLG1)* |
| reactive oxygen species metabolic process | 0.00696514 | 2.15707015 | *2(AKT1、STAT3)* |
| Signaling by Interleukins | 0.00847169 | 2.072029945 | *4(DLG1,AKT1,STAT3,TRAF6)* |
| Immune System | 0.011337868 | 1.945468604 | *6(DLG1、SEC22b、STAT3、TRAF6、AKT1、CTSL)* |
| Signaling by PDGF（platelet derived growth factor） | 0.01190797 | 1.924162268 | *2(STAT3、COL9A1)* |
| Prolactin signaling pathway | 0.0132978 | 1.876220203 | *2(AKT1、STAT3)* |
| Toll-like receptor signaling pathway | 0.0211029 | 1.675657859 | *2(AKT1、STAT3)* |
| HIF-1 signaling pathway | 0.0218329 | 1.660888574 | *2(AKT1、STAT3)* |
| Relaxin signaling pathway | 0.0267597 | 1.57251876 | *2(AKT1、GNAI1)* |
| metal ion transport | 0.02720879 | 1.565290771 | *2(DLG1、AKT1)* |
| MAPK signaling payhway | 0.02901 | 1.537452271 | *2(AKT1、TRAF6)* |

## Supplementary Table S5. Functional categories of genes in stop-lost

| stop_lost pathway | p-value | (-log10pvalue) | gene |
| --- | --- | --- | --- |
| MAPK | 1.03E-14 | 13.98716278 | *39TGFB1RASGRP1PDGFRBHRASMAPK8EGFRRAP1BINSRCACNA1DNTRK2RASGRF2NFKB1NFKB2MAPK9KDRTGFBR2IGF1RNTF3TRAF6HSPB1PRKCBCHUKEFNA1TNFFGF9FGF8PLA2G4AFGFR2FGFR3TAOK3KRASRPS6KA5TP53CASP3RPS6KA6INSBRAFCDC42ATF2* |
| Calcium signaling pathway | 5.58E-11 | 10.2533658 | *27 PDGFRB ATP2A2 LAP3 EGFR ADCY9 TNNC2 EDNRB CALM2 ADRB1 SPR CACNA1D ORAI1 CCKAR PRKCB MYLK3 PHKA2 PLCD4 ATP2B1 ATP2B2 TACR1 ADRA1A TPCN2 MYLK F2R ADRB2 CALMP LN* |
| Inflammation mediated by chemokine and cytokine signaling pathway | 4.10E-10 | 9.387216143 | *26 MYH11 ACTA1 PTGS2 LAP3 GNAI1 MYH3 MYH9 RGS4 NFKB2 ITGB1 SHC1 PRKCB MYLK3 CHUK PLCD4 RHOC PLA2G4A KRAS CXCL8 COL14A1 MYLK COL6A1 COL6A3 PAK3 COL12A1 CDC42* |
| mTOR signaling pathway | 1.70E-09 | 8.769551079 | *22 HRAS INSR MIOS INS FZD1 FZD3 FZD5 WDR24 CLIP1 RNF152 IGF1R FNIP1 PRKCB CHUK TNF MLST8 KRAS SGK1 RPS6KA6 GSK3B BRAF STRADA* |
| Relaxin signaling pathway | 3.31E-09 | 8.480172006 | *20 GNAI1 TGFB1 SHC1 SRC GNB1 HRAS MMP13 EGFR ADCY9 SMAD2 SMAD3 CREB3L2 KRAS NFKB1 EDNRB MAPK9 MMP2 MAPK8 TGFBR2 ATF2* |
| Prolactin signaling pathway | 5.65E-08 | 7.247951552 | *14 SRC SHC1 ESR1 PRLR HRAS CCND2 GSK3B CYP17A1 CISH INS NFKB1 MAPK8 MAPK9 KRAS* |
| Heterotrimeric G-protein signaling pathway-Gi alpha and Gs alpha mediated pathway | 1.52E-07 | 6.818156412 | *19 KCNJ3 ADRA1A CALM2 CREB3L2 ADRB1 GNB1 CHRM4 DRD2 RAP1B GSK3B RGS4 ADCY9 ADRB2 CALM HTR1D ADRA2C PHKA2 RGS8 GNAI1* |
| PDGF signaling pathway | 2.01E-07 | 6.696803943 | *17 PDGFRB RPS6KA5 SHC1 RPS6 KA6HRAS PKN2 FLI1 CHUK SRGAP2 FEV ARHGAP15 MAPK6 GSK3B JAK1 MAPK8 PIK3C3 BRAF* |
| Autophagy | 3.00E-07 | 6.522878745 | *17 MTMR4 IGF1R PIK3 C3GABARAPL1 TRAF6 RAB33B MLST8 HRAS CTSD HIF1A INS ATG7 LAMP1 ATG2A MAPK8 MAPK9 KRAS* |
| Aldosterone-regulated sodium reabsorption | 5.42E-07 | 6.266000713 | *10 NR3C2 SGK1 PRKCB ATP1B3 INS INSR ATP1A3 ATP1A2 ATP1A1 KRAS* |
| VEGF signaling pathway | 1.50E-05 | 4.823908741 | *10 SRC PTK2 HSPB1 HRAS PRKCB PTGS2 PLA2G4A CDC42 KDR KRAS* |
| Vascular smooth muscle contraction | 2.76E-05 | 4.559090918 | *14 ADRA1A MYH11 NPR2 CALM2 PRKCB CACNA1D ADCY9 MYLK MYL6 KCNMA1 BRAF MYLK3 PLA2G4A CALM* |
| Proximal tubule bicarbonate reclamation | 1.29E-04 | 3.88941029 | *6 PCK1 ATP1B3 SLC4A4 ATP1A3 ATP1A2 ATP1A1* |
| HIF-1 signaling pathway | 3.04E-04 | 3.517126416 | *11 IGF1R PRKCB EGFR HIF1A INS IL6 INSR SLC2A1 NFKB1 RBX1 LDHA* |
| Toll-like receptor signaling pathway | 2.65E-03 | 2.576754126 | *9 TBK1 TRAF6 CXCL8 CHUK IL6 TNF NFKB1 MAPK8 MAPK9* |
| Cortisol synthesis and secretion | 2.77E-03 | 2.557520231 | *7 ORAI1 CACNA1D ADCY9 CREB3L2 CYP17A1 CYP11A1 ATF2* |
| Cortocotropin releasing factor receptor signaling pathway | 1.40E-02 | 1.853871964 | *4 GNB1 CRH CRHR2 CRHR1* |

## Supplementary Table S6. Functional categories of genes in stop-gained

| stop_gain pathway | p-value | (-log10pvalue) | gene |
| --- | --- | --- | --- |
| MAPK signaling pathway | 4.80E-11 | 10.31875876 | *33 PDGFRB HSC71 CSF1R RAP1B INSR FLT4 FLT1 FGF3 CACNA1D NTRK1 NTRK2 RASGRF2 DUSP4 MAPK8 FGFR1 FGF17 TGFBR2 PDGFA HSPB1 PRKCB CHUK STK4 NFKB1 PLA2G4A FGFR2 TAOK2 TAOK3 TP53 CASP3 RPS6KA6 TIE2 ATF2 CDC42* |
| PDGF signaling pathway | 2.18E-09 | 8.661543506 | *20 PDGFA STAT3 SHC1 RPS6 KA6PDGFRB PKN2 FLI1 CHUK ETS1 SRGAP2 PIK3R5 ARHGAP15 JAK2 ARHGAP26 PIK3 MAPK6 ARHGAP6 GSK3B MAPK8 JAK1* |
| Oxytocin signaling pathway | 3.07E-06 | 5.512861625 | *17 KCNJ3 KCNJ12 NPR2 KCNJ2 CAMK2D PRKCB CACNA1D ADCY9 MYLK PIK3R5 CALM MYLK3 PIK3 PLA2G4A PPP1R12A EEF2 GNAI1* |
| mTOR signaling pathway | 1.09E-05 | 4.962573502 | *16 FLCN PRKCB ATP6V1A SGK1 RPS6 KA6WNT2 WNT1 STK11 CHUK GSK3B INSR CLIP1 WNT9A MIOS WNT5B STRADA* |
| HIF-1 signaling pathway | 9.00E-05 | 4.045757491 | *12 STAT3 CAMK2D PRKCB HIF1A IL6 INSR TFRC SLC2A1 TIE2 NFKB1 EP FLT1* |
| Vascular smooth muscle contraction | 1.16E-04 | 3.935542011 | *13 KCNMB1 MYH11 NPR2 CALCRL PRKCB CACNA1D ADCY9 MYLK KCNMA1 CALM MYLK3 PLA2G4A PPP1R12A* |
| Mineral absorption | 2.73E-04 | 3.563837353 | *8 SLC40A1 ATP1B3 ATP1A3 ATP1A2 ATP1A1 ATP2B1 SLC26A9 SLC46A1* |
| VEGF signaling pathway | 5.41E-04 | 3.266802735 | *8 PTK2 HSPB1 PRKCB HIF1A CRYAA ETS1 PIK3 PLA2G4A* |
| Autophagy | 9.59E-04 | 3.018181393 | *11 WIPI1 CTSL PPP2CA STK11 HIF1A ATG4C ATG2A GABARAPL1 MAPK8 MTMR4 EIF2S1* |

## Supplementary Table S7. Model based evolutionary analysis of positive selection by CodeML method

| Model | Parameter estimates | 2*ΔlnL* | Model compared | LRT p-value | Positive selection sites |
| --- | --- | --- | --- | --- | --- |
| M3 | *ω*0=0.40222,*ω*1=1.48614,*ω*2=9.66511, | 740.2631 | M0*vs.*M3 | 0 | - |
|  | (p0=0.43343,p1=0.32024,p2=0.24633) |  |  |  |  |
| M0 | *ω*0=0.95098 |  |  |  | Not alowed |
| M2a | *ω*0=0.30656,*ω*1=1.00000,*ω*2=7.55309, | 366.7956 | M1a*vs.*M2a | 0.001044622 | - |
|  | (p0=0.30383,p1=0.41677,p2=0.27940) |  |  |  |  |
| M1a | *ω*0=0.20267,*ω*1=1.00000, |  |  |  | Not allowed |
|  | (p0=0.44192,p1=0.55808) |  |  |  |  |
| M8 | p0=0.72025,p1=0.27975, p=0.48858, | 377.8723 | M7*vs.*M8 | 0.001334754 | * |
|  | q=0.17209, *ω=*7.87408 |  |  |  |  |
| M7 | p=0.10105, q=0.02430 |  |  |  | Not allowed |
| M8a | p0=0.44514, p1=0.55486, p=25.57944 | 365.0119 | M8a*vs.*M8 | 0.000120246 | Not allowed |
|  | q=99.00000,*ω=*1.00000 |  |  |  |  |
| *3 H 0.996** 4 S 0.996** 6 K 0.981* 9 T 1.000** 11 S 0.997** 14 S 0.982* 15 V 0.999** 16 L 0.996** 362 I 0.983* 370 K 1.000** 371 A 0.975* 372 V 0.992** 373 C 0.999** 375 Q 0.993** 376 L 0.999** 377 S 0.998** 386 T 1.000** 390 G 0.968* 391 Q 0.999** 400 L 0.990** 403 L 0.999** 405 H 0.995** 407 G 0.997** 409 E 0.999** 410 C 0.993** 412 L 0.975* 414 G 0.994** 415 G 1.000** 419 G 0.999** 421 P 0.986* 422 K 0.998** 424 V 0.975* 429 F 0.989* 432 L 0.980* 434 F 0.975* 436 V 0.966* 438 L 0.997** 445 T 0.997** 447 A 0.999** 449 V 0.998** 450 N 0.999** 452 I 1.000** 453 I 0.990* 455 Q 1.000** 456 R 0.998** 457 I 1.000** 461 K 1.000** 463 N 1.000** 464 I 0.999** 466 V 0.991** 467 S 0.999** 468 V 0.999** 470 S 1.000** 471 Y 0.990* 472 S 1.000** 473 F 0.996** 474 L 0.977* 475 K 1.000** 477 L 0.977* 478 D 0.989* 479 M 0.985* 481 I 0.985* 483 M 0.967* 484 R 0.995** 485 P 0.994** 486 V 0.993** 487 L 0.994** 489 P 0.980* 490 T 0.971* 492 Y 1.000** 493 I 0.996** 495 W 0.999** 496 S 0.998** 497 T 0.999** 498 I 0.999** 499 Y 0.998** 500 S 0.990* 502 T 1.000** 504 F 0.985* 505 F 0.998** 507 V 0.992** 509 V 1.000** 512 S 0.958* 513 A 0.988* 514 F 0.999** 515 M 0.997** 517 N 0.983* 518 C 0.998** 519 D 1.000** 520 I 0.997** 522 M 0.975* 523 M 0.987* 524 P 0.989* 526 Q 1.000** 527 L 0.975* 529 Q 0.995** 530 T 0.999** 531 R 0.999** 532 D 0.993** 533 P 0.997** 534 C 1.000** 535 G 1.000** | | | | | |
| **NOTE:** purified selection (p0), neutral selection (p1), positive selection (p2), p and q were the β distribution parameter. | | | | | |
| 2ΔlnL: twice the difference in log-likelihood values b. | | | | | |

## Supplementary Table S8. Sites found to be under positive selection by MEME and FEL methods

| MEME | P-value | FEL | P-value |
| --- | --- | --- | --- |
| 112 | 0.02 | 112 | 0.029 |
| 127 | 0.04 | 139 | 0.048 |
| 139 | 0.03 | 260 | 0.041 |
| 324 | 0.03 | 1122 | 0.008 |
| 342 | 0.03 | 1266 | 0.007 |
| 404 | 0.04 | 1274 | 0.011 |
| 455 | 0.02 | 1408 | 0.042 |
| 466 | 0.02 | 1414 | 0.006 |
| 561 | 0.02 | 1452 | 0.027 |
| 567 | 0.02 | 1460 | 0.037 |
| 643 | 0 | 1519 | 0.028 |
| 651 | 0.05 | 1575 | 0.016 |
| 654 | 0.01 | 1580 | 0.042 |
| 667 | 0.03 | 1583 | 0.042 |
| 702 | 0.02 | 1630 | 0.038 |
| 709 | 0.02 | 1651 | 0.037 |
| 710 | 0 | 1689 | 0.027 |
| 741 | 0.03 | 2655 | 0.048 |
| 745 | 0.03 |  |  |
| 766 | 0.02 |  |  |
| 936 | 0.01 |  |  |
| 971 | 0 |  |  |
| 973 | 0.04 |  |  |
| 1013 | 0.03 |  |  |
| 1019 | 0.04 |  |  |
| 1544 | 0.05 |  |  |
| 2118 | 0.05 |  |  |
| 2480 | 0.04 |  |  |
